# Congenital absence of infrarenal inferior vena cava and deep veins of the lower limbs: a case report

**DOI:** 10.1186/s13256-016-1015-3

**Published:** 2016-08-10

**Authors:** Abhishek Gupta, Sanjeev Kumar, Shyam S. Kothari

**Affiliations:** 1 Department of Cardiology, All India Institute of Medical Sciences, New Delhi, 110029 India; 2 Cardiac Radiology, All India Institute of Medical Sciences, New Delhi, 110029 India

**Keywords:** Inferior vena cava, Thrombosis, Congenital anomaly, Vein

## Abstract

**Background:**

Congenital anomalies of the venous system are known but congenital absence of infrarenal inferior vena cava with absent deep venous system of the lower limbs is extremely rare.

**Case presentation:**

We report the case of an 11-year-old Indian girl who presented with large venous collaterals on her anterior abdominal wall and recurrent non-healing venous ulcers on her left leg with complete absence of infrarenal inferior vena cava and absent deep veins of her lower limbs.

**Conclusions:**

Congenital absence of infrarenal inferior vena cava may occur with absence of the deep venous system of the lower limbs. We have reported this case because of its extreme rarity and to enhance awareness of this entity that has no treatment currently.

## Background

Anomalies of the venous system are not uncommon. Nearly 60 distinct inferior vena caval anomalies have been described in the literature [1]. Inferior vena cava (IVC) interruption with azygos continuation is a well-recognized anomaly that can be found in asymptomatic patients [1]. However, congenital absence of infrarenal IVC with absent deep venous system of the lower extremities is an extremely rare condition that may be associated with significant clinical manifestations. Here we report the case of an Indian girl who had large venous collaterals on her abdomen and recurrent non-healing venous ulcers on her left leg. On investigation, she had complete absence of infrarenal IVC and absent deep veins of her lower limbs.

## Case presentation

An 11-year-old Indian girl presented with a history of recurrent non-healing left leg venous ulcers associated with swelling of the involved limb. There was no associated erythema and her leg was non-tender. There was no history of trauma. There was no significant antenatal history in her mother and our patient was delivered normally at a local hospital with an uneventful postnatal period. There was no history of umbilical cannulation, cardiac catheterization, or any other femoral intervention. She had reported these symptoms since early childhood but no medical evaluation was done in the past. There were no other complaints.

**Fig. 1:**
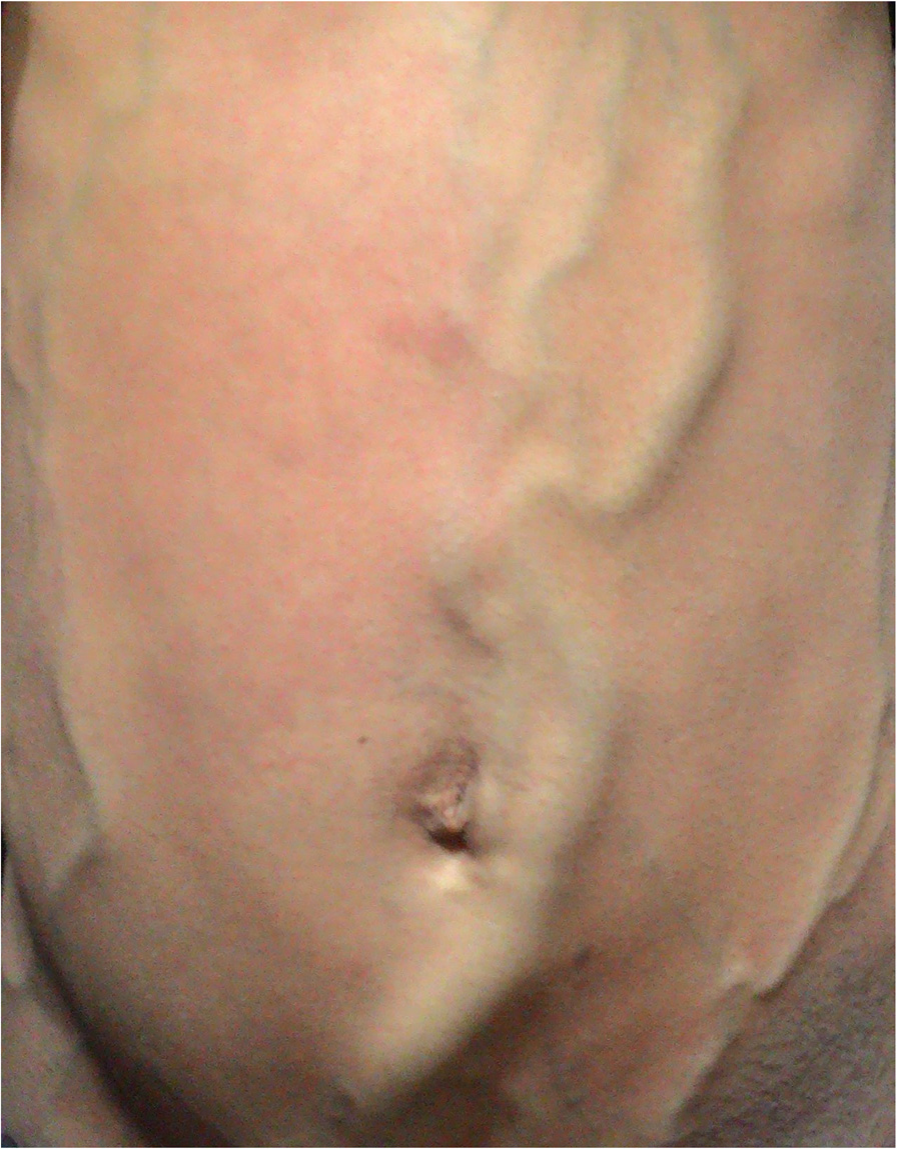
Large superficial collaterals on anterior abdominal wall

**Fig. 2:**
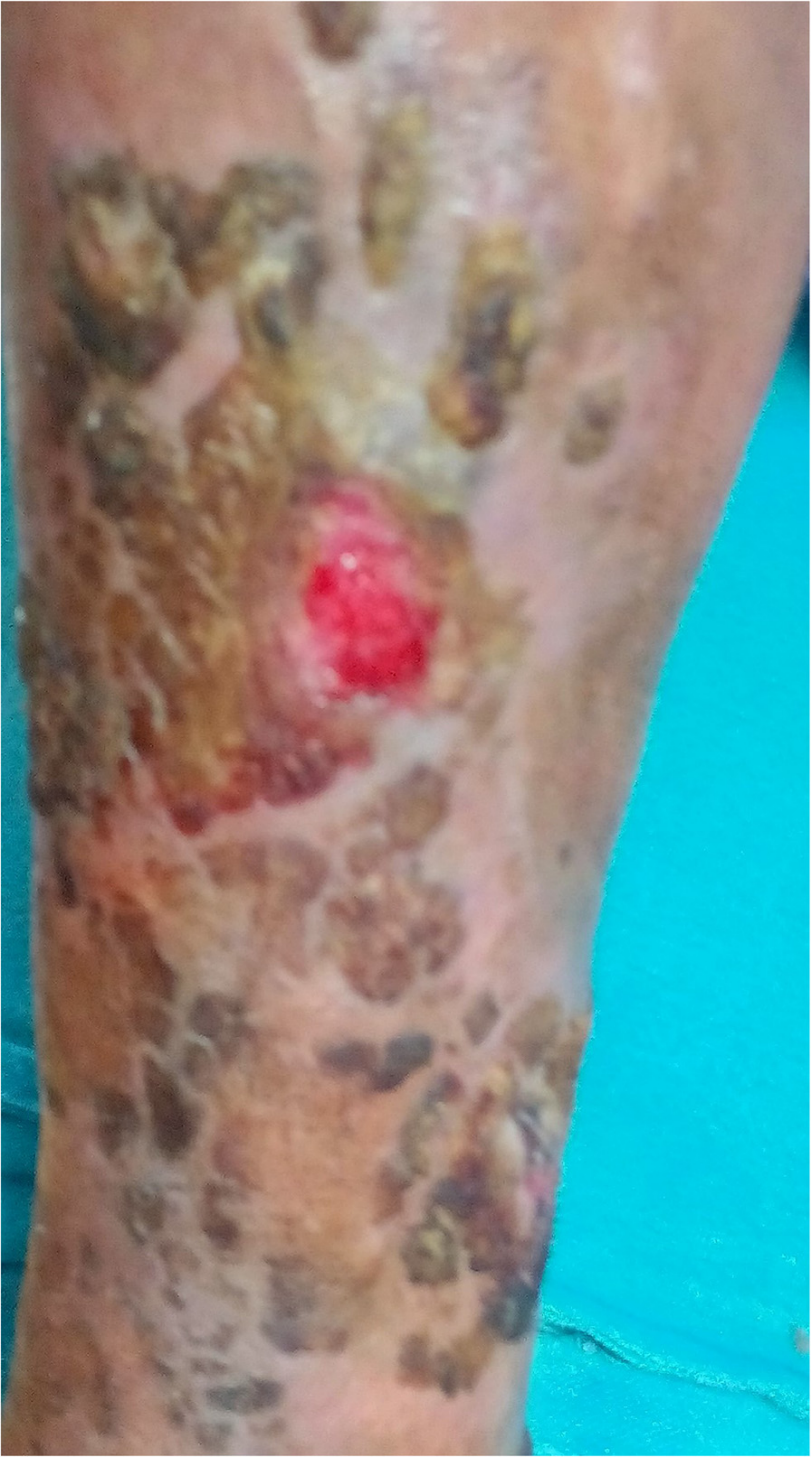
Non-healing venous ulcer on the left leg

Her physical examination was remarkable for large venous collaterals on her anterior abdominal wall with flow from below upwards (Fig. 1). She was also noted to have swelling and multiple venous ulcers on her left lower limb (Fig. 2). Both lower limbs had varicose veins. There was no calf tenderness. There was pedal edema on her left lower limb. The rest of her examination was normal.

On color Doppler examination, her infrarenal IVC as well as her bilateral internal and external iliac veins were not visualized. A short segment of her proximal right common femoral vein and proximal superficial femoral vein were faintly visualized. Her left-sided common femoral, superficial femoral, and popliteal veins were not visualized.

**Fig. 3:**
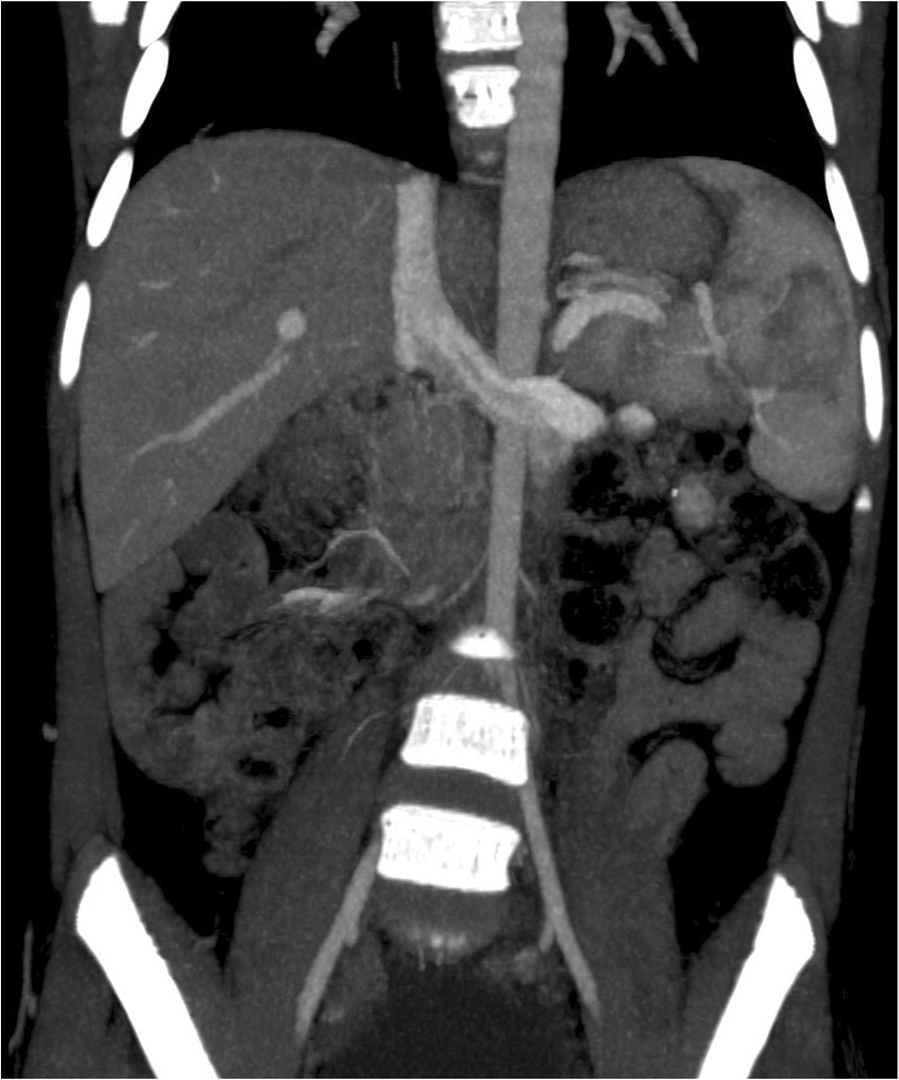
Computed tomography venogram (coronal section) showing normal drainage of renal vein into inferior vena cava with complete absence of infrarenal inferior vena cava

**Fig. 4:**
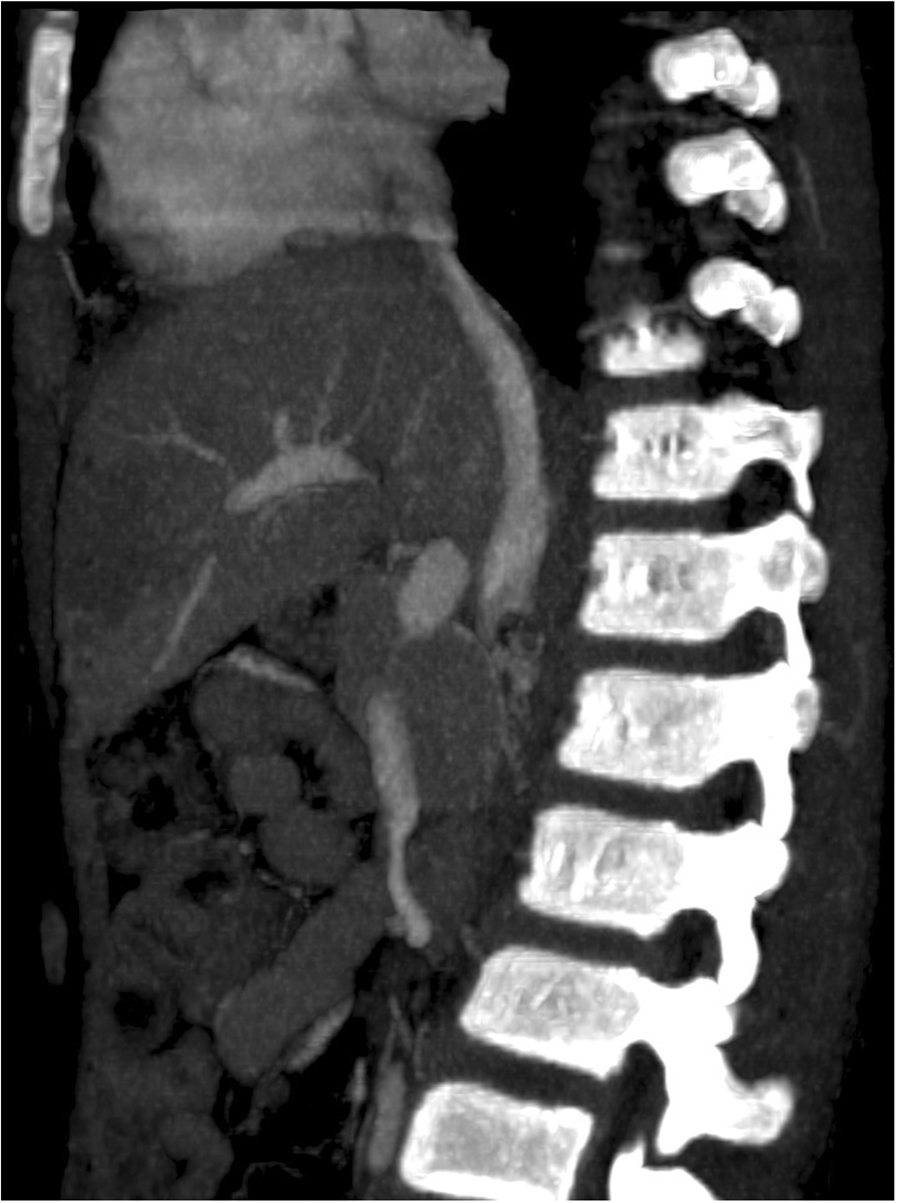
Computed tomography venogram (sagittal section) showing complete absence of infrarenal inferior vena cava and normal drainage of hepatic inferior vena cava into right atrium of her heart

A computed tomography (CT) venogram done to define her venous anatomy showed absence of infrarenal IVC as well as absence of bilateral common iliac and left common femoral veins (Fig. 3). A short segment of her right common femoral vein was seen with collaterals draining into her anterior abdominal wall. Her venous system at the level of the renal vein and above was normally developed (Fig. 4). There were well-developed collaterals over her anterior abdominal wall and in her bilateral lower limbs. The superficial venous system of her bilateral lower limbs was well developed and draining from collaterals.

Echocardiography showed normal cardiac anatomy and function.

With symptomatic treatment, her venous ulcers improved. There were no surgical or interventional therapies possible in this case. She was not started on anticoagulation therapy.

## Discussion

Congenital anomalies of the IVC are seen in 0.5 to 3 % of the general population and are mostly asymptomatic [1]. However, congenital absence of IVC is exceedingly rare and occurs with an estimated prevalence of 0.0005 to 0.001 % [2], although no systematic data are available. Most of the reports highlighted the absence of IVC; the absence of a deep venous system with an absence of IVC is even rarer.

Patients with congenital absence of IVC usually present with deep venous thrombosis (DVT), or recurrent non-healing venous ulcers [3–7]. The age of presentation is variable.

The genesis of this abnormality is speculative. D’Archambeau *et al*. [8] and Milner and Marchan [9] have proposed that absence of the infrarenal IVC can be a result of intrauterine or perinatal thrombosis of the IVC and not a developmental defect. A report by McDonald *et al*. [10] seemed to support this concept. They reported on a series of ten patients with renal vein thrombosis out of which seven were found during the perinatal period. In three of these patients, a thrombus was seen in the IVC in the initial venograms but on subsequent venograms the IVC could not be seen and was thought to be completely obstructed [10]. Another adolescent presenting with DVT and absent IVC had documented perinatal IVC thrombosis [11]. These reports clearly indicate that at least in some patients, a perinatal thrombosis might be the cause of absent IVC detected later in life. However, the additional absence of deep venous systems in our patient seems to indicate some other embryological insult responsible for the disease.

Others have suggested that absent IVC could be a developmental defect of IVC. The normal IVC develops from four segments; each segment is derived from different embryonic veins. The infrarenal segment develops from the right supracardinal vein and abnormal regression or persistence of the supracardinal vein results in various anomalies of the IVC [12]. As suggested by Swaiman *et al*., a late insult may lead to absence of infrarenal IVC as the infrarenal part of IVC is the last to develop, between the sixth and eighth week of life [13]. However, it is difficult to identify a single embryologic event that could fully explain this anomaly [3]. Superficial and deep venous systems are developmentally different with superficial veins appearing before the deep veins [14]. The superficial venous system was well developed in our patient and she had varicose veins due to the absence of IVC and undeveloped deep venous systems.

Chronic venous hypertension and varicosities leading to venous ulcers have been reported in patients with absent IVC that could be surgically bypassed [12]; however, the absence of iliofemoral veins precluded any bypass procedure in our patient. Massive serpiginous collaterals in patients with absent IVC might masquerade as a paraspinal mass [9].

In patients presenting with DVT and congenital IVC absence, lifelong anticoagulation therapy is recommended [15]. Our patient is currently on conservative management, whether vascular endothelial growth factor (VEGF) [16] or similar growth factors have any therapeutic role in such a patient remains to be seen.

## Conclusions

In conclusion, we report an extremely rare case of absence of IVC and absence of deep venous system of the lower limbs. Such patients may present with venous thrombosis or non-healing ulcers. This type of anomaly has no surgical or interventional treatment at present.

## Abbreviations

CT, computed tomography; DVT, deep venous thrombosis; IVC, inferior vena cava; VEGF, vascular endothelial growth factor
